# Plasma biomarkers associated with deployment trauma and its consequences in post-9/11 era veterans: initial findings from the TRACTS longitudinal cohort

**DOI:** 10.1038/s41398-022-01853-w

**Published:** 2022-02-26

**Authors:** Meghan E. Pierce, Jasmeet Hayes, Bertrand Russell Huber, Andreas Jeromin, Catherine B. Fortier, Jennifer R. Fonda, Heather Lasseter, Lauren Chaby, Regina McGlinchey, William Milberg

**Affiliations:** 1 Translational Research Center for TBI and Stress Disorders (TRACTS), VA Boston Healthcare System, Boston, MA USA; 2 Department of Psychiatry, Harvard Medical School, Boston, MA USA; 3 Department of Psychiatry, Boston University School of Medicine, Boston, MA USA; 4 Department of Psychology, The Ohio State University, 225 Psychology Building, 1835 Neil Avenue, Columbus, OH 43210 United States; 5 Chronic Brain Injury Initiative, The Ohio State University, 203 Bricker Hall, 190 North Oval Mall, Columbus, OH 43210 USA; 6 Cohen Veterans Bioscience, 535 8th Avenue, 12th Floor, New York, NY 10018 USA; 7 Geriatric Research, Educational and Clinical Center (GRECC), VA Boston Healthcare System, Boston, MA USA; 8 Present Address: Virgo Health 9th Floor 909 Third Avenue, New York, NY 10022 USA; 9 Present Address: Moderna 200 Technology Square, Cambridge, MA 02139 USA

**Keywords:** Biomarkers, Diagnostic markers

## Abstract

Mild traumatic brain injury (mTBI) is among the most common injuries sustained by post-9/11 veterans; however, these injuries often occur within the context of psychological trauma. Blast exposure, even in the absence of a diagnosable TBI, leads to changes in neural connectivity and congitive functioning. Therefore, considering clinical comorbidities and injury characteristics is critical to understanding the long-term effects of mTBI. Research is moving towards identifying diagnostic and prognostic blood-based biomarkers for TBI; however, few studies include other prevalent clinical and medical comorbidities related to deployment. Here, we present the initial cross-sectional relationships between plasma biomarkers, clinical, and medical comorbidities in a well-characterized longitudinal sample of 550 post-9/11 veteran men and women. We examined biomarkers associated with inflammation (interleukin 6 and 10, tumor necrosis factor α, and eotaxin) and neurodegeneration (neurofilament light, glial fibrillary acidic protein (GFAP), tau, brain derived neurotrophic factor, amyloid ß 40 and 42, phosphorylated neurofilament heavy chain, and neuron specific enolase). Univariate analyses of covariance (ANCOVA) were conducted to determine mean level differences between close blast (blasts that occur within 0–10 meters) and mTBI groups. Our primary findings were twofold: (1) Inflammatory markers were consistently higher in participants exposed to close blasts and were strongly related to deployment-related psychopathology. (2) GFAP was consistently lower in participants exposed to blast and mTBI and lower GFAP was associated with more severe psychological symptoms. More research is clearly needed; however, our findings indicate that chronic increased inflammation and decreased GFAP may be related to close blast exposure.

## Introduction

Approximately 20% post-9/11 active duty and veteran service members have reported at least one mild traumatic brain injury (mTBI), with more than one-third of these coming from blast-related sources such as improvised explosive devices (IEDs) and rocket propelled grenades RPGs [1]. Previous work suggests that mTBI, particularly those that are blast-related, are associated with long term cognitive and brain alterations [2–4]. Further, blast exposure, even in the absence of diagnosable mTBI, is associated with disruptions in functional brain connectivity [3] and deficits in cognitive functioning [2]. Work conducted by our group suggests a distinction between blast and clinical concussion. For example, Grande and colleagues [2] found that exposure to close blasts (within 10 meters), but not the number of lifetime concussions, was associated with poor memory performance. Robinson and colleagues [5] found that uptake of a positron emission tomography (PET) tracer that binds to tau proteins was related to blast exposure but not blunt concussion. Therefore it is critical to include blast exposure when examining the differential contributions of mTBI and psychopathology to overall functional outcome. Furthermore, it is becoming increasingly clear that mTBI rarely occurs outside of the context of other deployment-related comorbidities such posttraumatic stress disorder (PTSD) and depression. Thus, deep clinical characterization of deployment-related comorbidities is necessary to understand the long-term effects and identify diagnostic and prognostic plasma biomarkers of mTBI in veterans. The mechanism of mTBI and the presence of clinical comorbidities must be considered when treating veteran populations.

There is growing recognition that blood-based biomarkers may be useful in characterizing brain injury. Blood-based biomarkers have advantages over other biomarker methods such as magnetic resonance imaging (MRI) and PET because they are less invasive and are more time and cost effective. Recent advances in assay development, such as single molecule array technology (Simoa; Quanterix, Billerica, MA), has made it possible to detect markers that were previously undetectable in blood. In a cross-platform and cross-assay evaluation of five independent technologies in assessing four inflammatory cytokines, Simoa consistently demonstrated the highest sensitivity and precision [6].

Recent work using Simoa technology has demonstrated relationships between mTBI and blood-based biomarkers related to neuronal and glial degeneration [7–9]. Shahim and colleagues [9] investigated the relationship between serum neurofilament light (NfL), glial fibrillary acidic protein (GFAP), tau, and ubiquitin carboxyl-terminal hydrolase-L1 (UCH-L1) and TBI in a cohort of otherwise psychologically and physically healthy individuals over a longitudinal period of five years. The TBI cohort consisted of mostly blunt-related TBI, with only 5% of the sample having reported a blast-related TBI. In this cohort, NfL was found to be the best diagnostic and prognostic marker of TBI over the longitudinal period. Similarly, Shahim and colleagues [8] examined the effects of blunt-related TBI in athletes over a longitudinal period of five years, reporting that NfL remained significantly elevated in athletes with TBI compared to controls. Another recent study [7] examined inflammatory and neurological markers in older veterans with TBI living in residential retirement facilities. This group found that interleukin-6 (IL-6) and NfL distinguished veterans with TBI from control veterans. In veterans with TBI who were also cognitively impaired, GFAP concentrations were increased in comparison to controls. Taken together, these studies provide support for using blood biomarkers to identify mTBI; however, several questions remain that limit our current understanding of the biochemical correlates of mTBI. For instance, few prior studies have considered medical and psychiatric comorbidities that often co-occur with mTBI and are also known to influence biomarker levels. In addition, few studies have examined the extent to which various mechanisms of injury, such as blunt injury versus blast exposure, may influence blood biomarker levels.

Here we report data from the Translational Research Center for TBI and Stress Disorders (TRACTS), a large multidimensional longitudinal study that investigates the effects of psychological and physical trauma in active-duty service members and veterans over time. In this hypothesis generating report, we examined cross-sectional relationships between chronic mTBI of varying mechanisms, blast exposure, psychiatric and health variables, and 12 plasma-based biomarkers (see Supplementary Table 1 for a brief description of biomarker type and function). Biomarkers related to neuronal degeneration, axonal injury, and glial injury were chosen to investigate the long-term impacts of blast exposure and mTBI. Inflammatory biomarkers were chosen to investigate chronic inflammation related to psychological and brain trauma. Brain derived neurotropic factor (BDNF) was chosen based on its association with brain injury both in regulating neurodegeneration and neural protection [10–12] and in recovery [13, 14]. We hypothesized that close blast exposure and mTBI would be related to increases in pro-inflammatory markers, as measured by IL-6, TNFα, and eotaxin-1, and decreases in anti-inflammatory markers, such as interleukin-10 (IL-10). We also hypothesized that mTBI would be associated with an increase in biomarkers indicative of (i) glial degeneration, as measured by GFAP; (ii) neuronal degeneration as measured by amyloid beta 40 and 42 (Aß40, Aß42), neuron specific enolase (NSE), and total tau; and (iii) axonal damage, as measured by NfL and phosphorylated neurofilament heavy chain (pNF-H). Finally, we hypothesize that mTBI and blast exposure would be related to decreased concentrations of neuro-regenerative biomarker, BDNF.

## Materials and methods

### Participants

Participants were men (*n* = 496) and women (*n* = 54) consecutively enrolled post-9/11 veterans from TRACTS, VA Boston Healthcare System, a TBI National Network Research Center (see ref. [15] for a detailed descriptions of the methods). Supplementary Fig. 1 provides a description of how many participants were excluded based on a priori exclusion criteria. Briefly, exclusion criteria included prior serious medical and/or neurological illnesses unrelated to TBI, active suicidal and/or homicidal ideation requiring intervention, or a current diagnosis of bipolar disorder or psychotic disorder (except psychosis not otherwise specified due to trauma-related hallucinations) according to the Diagnostic and Statistical Manual of Mental Disorders DSM [16], as well as moderate to severe TBI.

All participants completed an approximately 10-hour research session that consisted of psychiatric interviews, the completion of self-report psychological questionnaires, as well as biological and neuropsychological assessment. The study was approved by the VA Boston Healthcare System Institutional Review Board and all participants provided written informed consent. Participants were provided $210 for their time and travel costs.

### Plasma biomarkers

Participants were asked to fast from 10:00 pm the night before arriving to the study site. Informed consent was obtained between 7:00 am and 7:30 am and blood was drawn immediately thereafter. Plasma was collected in ethylenediaminetetraacetic acid (EDTA) tubes, centrifuged, and then immediately frozen and stored at −70 °C. Plasma samples remained frozen until assayed. Assays were conducted in batches to avoid multiple freeze-thaw cycles. Assays to measure plasma biomarker concentrations were run in duplicate using the ultra-sensitive Simoa technology (Quanterix, Billerica, MA). The samples were aliquoted into 96-well plates using the same template for each plate and were diluted according to the manufactures’ specifications. Different biomarker assays were conducted on different days, and on average, two plates were run per day. The lab technician was blinded to the clinical status of participants. The assays were run on the automated high-definition 1 (HD-1) analyzer (Quanterix, Billerica, MA). The sensitivity and precision of Simoa technology was demonstrated in a recent cross-platform comparison for inflammatory biomarkers [6]. Data from plasma samples excluded if the reported coefficients of variation (CV) between duplicate runs were over 20% and/or the measured analyte concentrations were below the lower limit of quantification (LLOQ). Supplementary Table 2 describes missing and excluded biomarker data and Supplementary Table 3 provides the average CV and LLOQ for each biomarker.

### Clinical measures

#### Demographics, combat exposure, TBI, and symptom validity

A demographics questionnaire was used to collect information pertaining to sex, ethnicity, education, and health history. Combat exposure and other war experiences were assessed using the Deployment Risk and Resilience Inventory DRRI [17].

A doctoral-level psychologist administered the Boston Assessment of TBI-Lifetime BAT-L [18], which assessed participants’ exposure to blast munitions and lifetime history (military and civilian) of mild, moderate, and severe TBI. Blast exposure was defined as any blast that occurred within 100 meters and close blast exposure was defined as any blast that occurred within 10 meters. When cued on distance recall, participants were provided real-world examples of distances. For example, the cue for close-range blast (within 10 meters) is “approximately 32 feet or the length of 2 parking spaces.” The BAT-L defines blunt- or blast-related mTBI as defined by loss of consciousness lasting less than 30 min, and by both posttraumatic amnesia and altered mental status lasting for a period of less than 24 h. Blunt-related mTBI was defined as any head injury caused by blunt force directly to the head and blast-related mTBI was defined as any injury caused by exposure to over pressurized blast within 100 meters. The BAT-L was administered to assess all TBIs that occurred pre-, during, and post-deployment.

#### Psychiatric disorders

A doctoral-level psychologist conducted all psychiatric assessments. To assess for a history of PTSD, the Clinician Administered PTSD Scale for the DSM-IV CAPS [19] was administered. Additionally, the non-patients edition of the Structured Clinical Interview for DSM-IV Axis I Disorders SCID [20–22] was used to assess mood disorders, anxiety disorders, substance use disorders, and to screen for psychotic disorders that were used for pre-enrollment exclusionary criteria. BAT-L, CAPS, and SCID diagnoses were reviewed weekly by at least three doctoral level psychologists for diagnostic consensus.

#### Sleep quality and pain

The Pittsburgh Sleep Quality Index PSQI; [23] was administered to assess sleep quality. The PSQI is a 19-question self-report measure that assesses seven components of sleep quality (Subjective Sleep Quality, Sleep Latency, Sleep Duration, Habitual Sleep Efficiency, Sleep Disturbances, Use of Sleep Medication, and Daytime Dysfunction). Component scores range from 0 (No Difficulty) to 3 (Severe Difficulty) and are summed to create a Global Score that ranges from 0 to 21. The Short Form McGill Pain Questionnaire SF-MPQ; [24, 25] was used to assess pain. The SF-MPQ consists of 15 sensory and affective descriptors of pain that are rated on a scale from 0 (None) to 3 (Severe). These pain descriptor ratings are summed to create a pain index rating that ranges from 0 to 45.

#### Medical and metabolic screening

Measures related to general and cardiometabolic health were collected by a medical technician. Participants completed a comprehensive medical background questionnaire that assessed health history (for detailed Methods see ref. [15]). Cardiometabolic health and symptom data collection included blood pressure, height, weight, waist-to-hip ratio, body mass index, and a blood draw. Blood testing included chemistry for fifteen markers of cardiovascular health, renal health, and general metabolism. Blood samples for C-Reactive Protein, glucose, HDL, LDL, triglycerides, homocysteine and A1C were processed immediately and shipped on the same day to Quest Diagnostics (Cambridge, MA) for quantification.

### Statistical analyses

Plasma biomarker data were not normally distributed and thus were log transformed before analysis. Means and standard deviations presented in our results section reflect the log transformed units. Data were considered outliers if the log transformed value was three standard deviations above the mean. Five tau samples met this criterion and were excluded from analyses. To ensure that plasma biomarker analyses included the full sample available, separate one-way analysis of covariance (ANCOVA) tests were conducted to investigate mean level group differences. Participants with plasma biomarker and/or clinical measures that were missing, outliers, or excluded were only excluded from analyses in which those specific measures were used. The Benjamini and Hochberg [26] correction method was used to control for the false discovery rate for comparisons within each plasma biomarker group (i.e. Neuroprotective, Anti-Inflammatory, Pro-Inflammatory, Neurodegeneration, Neural Damage, Glial Damage). Age, sex, and number of mTBIs or blast exposures were included as covariates in all mean-level analyses. The relationships between time since injury and plasma biomarkers were examined (see Supplementary Table 4); however, no significant relationships were revealed, and as such these variables were not included as covariates to avoid overfitting our models. Partial correlations were conducted to examine the relationships between clinical and medical comorbidity, symptom severity, and plasma biomarkers controlling for age and sex. All analyses were conducted using SPSS 27.0.1.0 software (IBM Corporation, Armonk, NY).

## Results

### Demographics and clinical characteristics

Table 1 summarizes demographic and clinical characteristics. Overall, the TRACTS sample is predominantly male (90.18%) with an average age of 33.34 years. Blast exposure was common in our sample, with 78.18% endorsing at least one blast exposure at any range (0–100 meters) and 42.54% experiencing at least one close blast. Furthermore, mTBI was also common in our sample, with 69.09% endorsing at least one mTBI at some point during their lifetime, 44.91% endorsing at least one mTBI (blast or blunt) during their military service, and 28.55% endorsing at least one blast-related mTBI. The mean time since the most recent mTBI occurred was 9.15 years (SD = 8.28) and ranged from 0 to 46 years. Similarly, the mean time since the most recent military blast was 6.50 years (SD = 3.82) and ranged from 0 to 27 years. Just under two-thirds of our sample (58.73%) had a current diagnosis of PTSD. Additionally, current mood disorders (27.27%) and anxiety disorders (18.73%) were prevalent in our sample.

**Table 1:** Demographics and clinical characteristics.

	*n* or *M*	% or SD	Range
Age	33.34	9.01	19–64
Men	496	90.18%	–
*Race*			
White, Non-Hispanic	401	72.91%	–
Black, Non-Hispanic	44	8.00%	–
Hispanic	77	14.00%	–
Native American	6	1.09%	–
Asian	21	3.82%	–
Pacific Islander	1	0.18%	–
*Education*			
High school or less	175	31.82%	–
Bachelor’s degree/ some college	331	60.18%	–
Advanced degree	44	8.00%	–
*Service branch*			–
Army	356	64.73%	–
Navy	27	4.91%	–
Marines	119	21.64%	–
Airforce	46	8.36%	–
Coast guard	2	0.36%	–
*Blast exposure & traumatic brain injury*			
0–10 Meters	2.62	19.66	0–416
11–25 Meters	2.91	11.30	0–204
26–100 Meters	23.62	92.44	0–999
Total blast exposed	430	78.18%	–
Lifetime TBI	380	69.09%	–
Military TBI	247	44.91%	–
Blast-related TBI	157	28.55%	–
Years since last TBI	9.15	8.28	0–46
Years since last Blast	6.50	3.82	0–27
*Clinical diagnoses*			
Current PTSD	323	58.73%	–
Current mood disorder	150	27.27%	–
Current anxiety disorder	103	18.73%	–
Current alcohol use disorder	71	12.91%	–
Current substance use disorder	17	3.09%	–

*n* = 550. Range refers to the range of reported incidents or scores per category or scale where a mean or standard deviation is reported.

### Close blast exposure

Our primary blast analysis focused on the difference between a history of close blast exposure and no history of blast exposure. Previous research from our center indicates that close blast exposure leads to disruption of brain networks [3, 27], white matter signal abnormalities [4, 28], and cognitive dysfunction [2] even in the absence of mTBI. However, we also provide supplementary analyses that examined distance from blast (Supplementary Table 5) and blast exposure at any distance compared to no blast exposure (Supplementary Table 6). Our main analysis consisted of an ANCOVA, where the plasma biomarker was the dependent variable, blast group consisting of two levels (No Blast and Close Blast) was the independent variable, and age, sex, and number of close blasts were the covariates. The Close Blast group (*n* = 234) included only those individuals with a history of at least one blast exposure within 10 meters; however, these individuals may have also experienced one or more blasts at any distance range. The No Blast group (*n* = 120) included individuals who reported no blast exposures within 100 meters.

Table 2 provides a summary of means, standard deviations, and corrected, and uncorrected significance values for ANCOVA tests for differences in log transformed plasma biomarkers (pg/mL) by close blast exposure groups, only the significant tests will be discussed here (see Supplementary Figs. 2 and 3 for visual representation of significant data and Supplementary Table 7 for analyses using raw data). Participants exposed to close blast had significantly higher levels of IL-6 (*F*(1, 290) = 6.41, *p* = 0.012, *q* = 0.036, η_p_^2^ = 0.022), TNFα (*F*(1, 291) = 4.28, *p* = 0.040, *q* = 0.040, η_p_^2^ = 0.014), and eotaxin (*F*(1, 305) = 5.13, *p* = 0.024, *q* = 0.036, η_p_^2^ = 0.017), compared to those with no blast exposure. Participants exposed to close blast had significantly lower levels of GFAP (*F*(1, 280) = 8.77, *p* = 0.003, η_p_^2^ = 0.030).

**Table 2:** Mean level differences in plasma biomarkers related to reported close blast exposure.

	No Blast *M* (SD)	Close Blast *M* (SD)	*p*-Value	*q*-Value
*Neuroprotection*				
BDNF	7.02 (1.18)	7.25 (1.08)	0.084	0.084
*Anti-Inflammatory*				
IL-10	−0.38 (0.51)	−0.31 (0.59)	0.401	0.401
*Pro-Inflammatory*				
IL-6	0.22 (0.54)	0.40 (0.72)	0.012	0.036
TNFa	0.93 (0.29)	1.02 (0.30)	0.040	0.040
Eotaxin	3.64 (0.36)	3.72 (0.35)	0.024	0.036
*Neurodegeneration*				
Aß40	5.36 (0.16)	5.34 (0.17)	0.861	0.874
Aß42	2.07 (0.17)	2.06 (0.22)	0.874	0.874
Tau	0.43 (0.58)	0.27 (0.55)	0.038	0.094
NSE	9.29 (0.43)	9.42 (0.49)	0.047	0.094
*Neuronal damage*				
NfL	1.73 (0.47)	1.62 (0.50)	0.201	0.201
pNF-H	3.10 (0.98)	3.23 (0.97)	0.178	0.201
*Glial damage*				
GFAP	4.22 (0.35)	4.06 (0.37)	0.003	0.003

No Blast (*n* = 120) refers to individuals with no history of blast exposure and Close Blast (*n* = 234) refers to individuals who have history of close blast exposure. *q-*Value represents the Benjamini–Hochberg correction for multiple comparisons. Means and standard deviations are reported as log transformed concentrations (pg/mL) after controlling for age, sex, and number of close blast exposures.

While NSE and tau did not meet the standards for statistical significance after correction for multiple comparisons (*p* > 0.05) it is important to note that the uncorrected *p*-values indicated a statistically significant difference (see Supplementary Fig. 4 for visual representation of data). Individuals with close blast exposure had higher concentrations of NSE (*F*(1, 292) = 3.98, *p* = 0.047, *q* = 0.094, η_p_^2^ = 0.013), and lower concentrations of total tau (*F*(1, 236) = 4.37, *p* = 0.038, *q* = 0.094, η_p_^2^ = 0.018) compared to individuals with no history of blast exposure.

When examining blast exposure at any range, we did find that GFAP was significantly lower in participants exposed to any blast compared to those not exposed to blast (*F*(1, 473) = 8.27, *p* = 0.004, η_p_^2^ = 0.019). Participants exposed to blast had significantly higher IL-6 concentrations compared to those with no blast history (*F*(1, 460) = 4.73, *p* = 0.030, η_p_^2^ = 0.010); however this result did not maintain significance after controlling for multiple comparisons. Supplementary Blast Distance analyses did not reveal any statistically significant differences beyond those reported between blast groups.

### Traumatic brain injury

When considering mTBI, we compared those with at least one military-related mTBI (*n* = 247) to those who did not have a military-related mTBI (*n* = 303). Military mTBI, rather than lifetime mTBI, was examined because mTBIs that occur within a military context often co-occur with a traumatic and/or blast events and are therefore of specific interest in the context of comorbidities. We also examined the mechanism of lifetime mTBI, which includes those participants with one or more mTBIs that occurred throughout the lifespan. The Non-mTBI group (*n* = 170) consisted of individuals who had no history of TBI. The Blunt mTBI group (*n* = 224) consisted of individuals who had sustained at least one blunt injury and may have experienced a blast exposure but not a blast-related mTBI, and the Blast mTBI group (*n* = 156) included individuals who had a history of at least one blast-related mTBI.

Table 3 displays the results for log transformed plasma biomarkers (pg/mL) across military mTBI and mTBI mechanism groups (Supplementary Tables 8 and 9 provide these results using the raw plasma data), only significant findings will be discussed. When considering mTBIs sustained during military service, participants with military mTBI had significantly lower GFAP concentrations than participants with no history of mTBI (*F* (1, 437) = 6.95, *p* = 0.009, η_p_^2^ = 0.016). When considering mTBI by mechanism of injury, participants GFAP was significantly different between groups (*F*(2, 436) = 4.40, *p* = 0.013, η_p_^2^ = 0.020). Pairwise comparisons revealed that GFAP was significantly lower in participants with blast-related mTBI compared to those with no history of mTBI (*p* = 0.007) and participants with blunt-related mTBI (*p* = 0.014).

**Table 3:** Mean level differences in plasma biomarkers related to military mTBI and mechanism of injury.

	Military TBI					Mechanism of TBI			
	Non-mTBI	mTBI	*p*-value	*q*-Value	No TBI	Blunt TBI	Blast TBI	*p*-value	*q*-Value
*Neuroprotection*									
BDNF	7.06 (1.16)	7.23 (1.13)	0.142	0.142	7.16 (1.26)	7.04 (1.08)	7.24 (1.12)	0.261	0.261
*Anti-Inflammatory*									
IL-10	−0.31 (0.67)	−0.34 (0.54)	0.631	0.631	−0.33 (0.64)	−0.33 (0.65)	−0.32 (0.53)	0.977	0.977
*Pro-Inflammatory*									
IL-6	0.32 (0.65)	0.39 (0.68)	0.151	0.453	0.31 (0.57)	0.30 (0.67)	0.46 (0.72)	0.025	0.075
TNFα	0.99 (0.33)	0.99 (0.33)	0.955	0.955	0.99 (0.33)	0.97 (0.33)	1.02 (0.32)	0.425	0.425
Eotaxin	3.69 (0.37)	3.73 (0.36)	0.364	0.546	3.71 (0.39)	3.68 (0.34)	3.74 (0.36)	0.358	0.425
*Neurodegeneration*									
Aß40	5.35 (0.22)	5.33 (0.18)	0.553	0.885	5.34 (0.23)	5.34 (0.20)	5.35 (0.17)	0.872	0.872
Aß42	2.07 (0.21)	2.07 (0.21)	0.886	0.886	2.08 (0.21)	2.05 (0.20)	2.08 (0.21)	0.368	0.822
Tau	0.35 (0.56)	0.32 (0.54)	0.664	0.885	0.33 (0.59)	0.36 (0.51)	0.29 (0.56)	0.617	0.822
NSE	9.35 (0.49)	9.39 (0.47)	0.270	0.885	9.34 (0.49)	9.34 (0.50)	9.44 (0.46)	0.575	0.822
*Neuronal damage*									
NfL	1.71 (0.47)	1.64 (0.52)	0.181	0.319	1.72 (0.44)	1.67 (0.50)	1.64 (0.53)	0.438	0.876
pNF-H	3.25 (0.99)	3.12 (0.96)	0.319	0.319	3.19 (0.96)	3.20 (1.02)	3.17 (0.95)	0.961	0.961
*Glial damage*									
GFAP	4.16 (0.36)	4.07 (0.37)	0.009*	0.009*	4.18 (0.39)	4.14 (0.33)	4.03 (0.37)	0.013	0.013

Military mTBI (*n* = 247) refers to individuals who experienced at least one mTBI during military service and Non-Military mTBI (*n* = 303) refers to individuals with no mTBI or those who experienced at least one mTBI pre- or post-deployment. No mTBI (*n* = 170) refers to individuals with no history of mTBI, Blunt mTBI (*n* = 224) refers to individuals who experienced at least one blunt-injury related mTBI, and Blast mTBI (*n* = 156) refers to individuals who have experienced at least one blast-injury related mTBI. *q-*Value represents the Benjamini–Hochberg correction for multiple comparisons. Means and standard deviations are reported as log transformed concentrations (pg/mL) after controlling for age, sex, and number of mTBIs.

Prior to corrections for multiple comparisons, IL-6 was significantly different between TBI mechanism groups, (*F*(1, 459) = 3.73, *p* = 0.025, *q* = 0.075, η_p_^2^ = 0.016), pairwise comparisons revealed that individuals with blast-related mTBI had higher IL-6 concentrations compared to those with no history of mTBI (*p* = 0.018) and blunt-related mTBI (*p* = 0.015). However, these differences were not statistically significant after correction for multiple comparisons.

### Correlations between plasma biomarkers and psychiatric and medical variables

Relationships between plasma biomarkers and clinical variables are displayed in Tables 4 and 5. Partial correlations were conducted to examine the relationship between psychiatric, medical, and plasma biomarkers, controlling for age and sex and corrected for multiple comparisons. Overall, increased inflammatory biomarker concentrations, particularly, IL-6, were associated with more severe psychopathology and worse functional outcomes. GFAP was negatively related to PTSD symptom severity, combat exposure, anxiety, stress, and alcohol use. In general, neurodegenerative markers were not related to psychological outcomes.

**Table 4:** Relationships between plasma biomarkers and psychiatric comorbidities.

	BDNF	IL-10	IL-6	TNFα	Eotaxin	Aß40	Aß42		Tau	NSE	NfL	pNF-H	GFAP
PTSD Symptom severity (CAPS)	0.042	−0.023	0.155***	0.062	0.066	−0.032		−0.031	−0.003	0.044	−0.043	−0.025	−0.100*
*DRRI Subscale total score*													
Combat	0.067	0.028	0.081	0.107*	0.105*	0.007		0.029	−0.065	0.083	−0.046	0.043	−0.186***
Other war experiences	0.070	−0.007	0.080	0.053	*0.112*	−0.011		−0.028	−0.086	0.031	−0.010	0.034	−0.120*
*Depression anxiety and stress scale*													
Anxiety total	0.050	0.012	*0.113*	0.082	0.061	−0.013		0.000	−0.035	0.036	−0.046	0.006	−0.123*
Depression total	0.048	0.112*	0.138*	0.092	0.074	−0.009		−0.032	−0.018	0.017	−0.046	−0.033	−0.057
Stress total	0.017	−0.008	0.105	0.043	0.031	−0.037		−0.047	−0.025	0.023	−0.043	0.029	−0.112*
*Lifetime Drinking History*													
Total (weight corrected)	0.031	0.057	0.065	0.117*	0.076	−0.001		−0.061	−0.088	0.048	−0.049	−0.011	−0.097*
Average drinks	−0.009	0.011	0.177***	0.132**	0.057	0.030		−0.024	−0.070	0.019	−0.061	−0.011	−0.083
Maximum drinks	0.034	0.017	0.157***	0.136**	0.057	−0.048		−0.060	−0.081	0.044	−0.061	−0.026	−0.090
SMAST Total lifetime score	0.034	0.059	0.057	0.058	0.023	−0.008		−0.139*	−0.152*	−0.039	−0.076	0.031	−0.054
*Fagerstrom test of nicotine dependence*													
Total cigarette score	−0.046	−0.005	−0.064	0.035	0.043	0.145		0.092	0.082	0.035	0.141	0.023	−0.158
Total chew score	−0.068	−0.025	0.027	0.017	0.043	0.099		0.102	−0.149	0.033	0.010	−0.006	−0.155
NSI: Total score	0.046	0.037	0.143**	0.116*	0.070	0.010		0.017	−0.061	0.039	−0.030	−0.013	−0.107*
WHODAS Complex total score	0.051	0.052	0.122*	0.103*	0.071	−0.047		−0.023	−0.058	0.031	−0.057	−0.053	−0.085
McGill: Current overall pain	−0.001	0.030	0.059	0.074	0.006	−0.011		−0.024	0.044	0.066	−0.067	−0.029	−0.102*
PSQI: Global score	0.078	−0.020	0.178***	0.112*	0.068	−0.012		0.026	−0.035	0.104*	0.003	0.045	−0.076

Partial correlations controlling for age and sex. *< 0.05; **< 0.01, ***< 0.001. All significance indicators reflect Benjamini–Hochberg corrected values.

**Table 5:** Relationships between plasma biomarkers and medical comorbidities.

	BDNF	IL-10	IL-6	TNFα	Eotaxin	Aß40	Aß42	Tau	NSE	NfL	pNF-H	GFAP
Age	0.101*	−0.011	0.074	0.048	0.122*	−0.077	−0.072	−0.123*	0.104*	0.348***	0.187***	0.277***
Mean arterial BP	0.211***	−0.061	0.078	−0.046	0.093	−0.073	−0.064	−0.002	0.217***	−0.171***	−0.019	−0.113*
Systolic BP	0.222***	−0.036	0.093	−0.037	0.089	−0.083	−0.079	−0.043	0.241***	−0.141**	0.009	−0.126**
Diastolic BP	0.174***	−0.070	0.057	−0.046	0.083	−0.057	−0.045	0.026	0.174***	−0.168***	−0.035	−0.088
Body mass index	0.083	−0.020	0.345***	0.198***	−0.038	−0.104*	−0.149**	−0.030	0.135**	−0.320***	−0.079	−0.218***
Waist to hip ratio	0.091	0.043	0.219***	0.214***	−0.036	0.009	−0.039	−0.029	0.136***	−0.194***	−0.001	−0.189***
Total cholesterol	0.087	−0.092	−0.086	−0.083	0.041	0.002	0.019	0.025	0.145**	−0.141**	−0.037	−0.081
HDL Cholesterol	−0.037	−0.149**	−0.184***	−0.270***	−0.010	−0.040	−0.055	0.012	−0.079	0.140**	0.085	0.094
LDL Cholesterol	0.086	−0.054	−0.017	−0.052	0.051	−0.054	−0.034	0.005	0.054	−0.159***	−0.090	−0.058
Triglycerides	0.075	0.027	0.021	0.127**	0.015	0.091	0.100*	0.055	0.215***	−0.056	0.010	−0.104*
Homocysteine	0.076	0.089	0.003	0.067	0.049	0.080	0.116*	−0.016	0.080	0.085	0.026	0.080
Glucose	0.127*	−0.016	0.042	0.020	0.069	−0.012	−0.028	−0.068	−0.010	0.085	0.040	−0.096*
A1C	0.093	−0.040	0.077	0.020	0.045	0.039	0.031	−0.045	0.027	0.051	0.037	−0.065
C-Reactive protein	0.011	0.186***	0.427***	0.341***	−0.034	0.040	0.036	0.047	−0.001	−0.113*	−0.009	−0.144**

Partial correlations controlling for age and sex. *< 0.05; **< 0.01, ***< 0.001. Partial correlation for age was corrected for sex. All significance indicators reflect Benjamini–Hochberg corrected values. BP refers to blood pressure, HDL refers to high-density lipoprotein and LDL refers to low-density lipoprotein.

Cardiometabolic health was assessed through several biological and physiological variables. In general, increased inflammation was related to maladaptive health outcomes. Markers of neuronal health showed differential relationships with health variables. BDNF, NSE, and NfL were the most robust in terms of number and strength of relationships to the health variables. BDNF was positively related to age, blood pressure, hip to waist ratio, and glucose. Overall, NfL and GFAP were negatively correlated with health variables, indicating worse outcomes.

## Discussion

The primary purpose of this hypothesis generating study was to present the initial relationships between blast exposure, mechanism of mTBI injury, and clinical comorbidities with plasma biomarkers relating to inflammation, neurodegeneration, and injury in a large cohort of post-9/11 veterans. This is the first study to examine a comprehensive panel of plasma biomarkers in a single cohort with a detailed characterization of mTBI, clinical, and health comorbidities. The main findings of these preliminary analyses were twofold.

First, inflammatory markers IL-6, TNFα, and eotaxin were significantly higher in participants with a history of close blast exposure compared to those who had no history blast exposure. Acute inflammation following blunt-related TBI is well-established and is predictive of negative outcomes [7, 29, 30]. Similar relationships have been found in rodent models of blast exposure [31, 32]; however, very few studies have investigated the chronic effects of blast exposure and inflammatory biomarkers in humans. One study [33] found an increase in IL-6 and TNFα directly following moderate blast exposure during training exercises; however, those levels returned to baseline within a day following exposure. Our findings provide evidence of chronic elevated inflammation following exposure to close blast. One important distinction between Gill and colleagues (2017) and our current cohort is that blast exposures that occurred during training were excluded in the current cohort unless safety protocols were not followed. It may be that chronic inflammation arises from a synergistic effect of psychiatric comorbidities during the blast. Indeed, all markers of inflammation were associated with maladaptive clinical outcomes (e.g., PTSD, alcohol use, etc.). The inflammatory cytokines, IL-6 and TNFα, were the most strongly associated with deployment-related pathology like more severe PTSD, problem drinking, increased neurobehavioral symptoms, and sleep dysfunction, as well as maladaptive functional outcomes. Thus, chronic inflammation may be a transdiagnostic marker of deployment-related pathology.

Next, GFAP was consistently lower in individuals who were blast exposed, regardless of blast distance, and those who had a history of mTBI, and was inversely related to combat exposure, alcohol use, and cigarette smoking. Blast exposure and blast-related mTBI showed the strongest effect in decreased GFAP. While these findings may conflict with recent research [7] where TBI was associated with higher GFAP concentrations, GFAP in CNS-derived “exosomes” were examined in previous work rather than in plasma. These results indicate that lower GFAP may be a unique feature of blast-related injury and may also be related to the chronicity of blast injury. It should be noted that the decrease between injury groups and control groups was modest and we cannot comment on the degree to which these levels lead to clinically significant change in metrics related to deployment trauma. However, we do find that lower GFAP is related to more severe psychopathology, including PTSD, depression, stress, neurobehavioral symptoms, and pain. Interestingly, blast-related attenuated GFAP was also observed in a recent study [34] which demonstrated decreased GFAP concentrations within one hour of blast exposure in the context of breacher training. Future research will examine the relationship between GFAP, neuroimaging correlates and clinical comorbidities to elucidate the underlying factors contributing to the lower GFAP concentrations in veterans with blast exposure and mTBI.

There are limitations to this study that should be considered when interpreting our findings. First, the data presented here are retrospective in nature. The clinical interviews and self-report measures require participants to report events that happened several months or years prior to the interview date. To reduce the impact of reporting bias, we conduct an in-depth semi-structured interview, developed by our center [18], to assess mTBI and blast exposure. The use of semi-structured interviews for identifying head injury is the gold standard in our field [35]. Furthermore, while the data presented here are cross-sectional, they are part of an ongoing longitudinal study. Examining longitudinal change in plasma biomarkers related to blast exposure and mTBI is important to understanding the long-term effects of injury. Next, we excluded individuals who sustained a moderate to severe TBI. It is possible that our null findings on biomarkers related to neural injury, particularly NfL, may be related to severity of injury. Finally, we did not have the statistical power to examine sex differences in mTBI, blast exposure, or plasma biomarkers. Supplementary Tables 10, 11, and 12 present raw unanalyzed mean level data from both men and women veterans. Although it is unanalyzed, it is clear that men and women follow similar patterns of increased or decreased biomarker by injury status. Future work should examine how biomarker profiles differ between the sexes.

Presented here are novel findings from the preliminary analysis of a large panel of plasma biomarkers in a well-characterized post-9/11 veteran cohort. While significant progress has been made in identifying prognostic and diagnostic markers of TBI [7–9], much more work is needed to fully characterize the neurobiological profile of TBI and predictors of recovery. Our team has several studies underway to extend our current approach. We are currently examining relationships between plasma biomarkers and brain morphometry and functional connectivity. We are also examining how mTBI in a multi-comorbidity sample may either confer risk for or accelerate the development of neurodegenerative diseases, such as Alzheimer’s disease. Future studies may focus on the use of a machine learning-based systems-biology approach to facilitate the development of biologically based classifications (biotypes) of these disorders and an understanding of common and unique pathways and mechanisms.

## Supplementary information

Supplemental Figure Legend

Supplemental Tables

Supplemental Figure 1

Supplemental Figure 2

Supplemental Figure 3

Supplemental Figure 4
