# Supplementary material for: Plasma biomarkers associated with deployment trauma and its consequences in post-9/11 era veterans: initial findings from the TRACTS longitudinal cohort
Source: Transl Psychiatry. 2022 Feb 26;12:80. doi: 10.1038/s41398-022-01853-w (PMC8881445; doi:10.1038/s41398-022-01853-w)
Supplement: Supplementary file 1 — Supplemental Figure Legend [file 41398_2022_1853_MOESM1_ESM.docx]

**Figure Legend**

**Supplemental Figure 1.**

Description of exclusion process.

**Supplemental Figure 2.**

Panel A. Results from significant ANCOVA analyses testing differences between blast groups on residualized values for log transformed (pg/mL) GFAP after controlling for age, sex, and number of close blasts. This data is also provided in Table 2. No Blast refers to participants with no history of blast (n = 120) and Close blast refers to participants who have exposure to blasts within 10 meters (n = 234). Significant values reflect Benjamini-Hochberg correction for multiple comparisons. Error bars reflect standard error.

Panel B. Box plot with scatter plot overlay represents log transformed, unadjusted data grouped by no blast and close blast exposure. Error bars reflect the 95% confidence interval.

**Supplemental Figure 3.**

Panel A. Results from significant ANCOVA analyses testing differences between blast and mTBI groups on residualized values for log transformed (pg/mL) IL6, TNFa, and Eotaxin after controlling for age, sex, and number of close blasts or number of mTBIs. This data is also provided in Table 2 and 3. No Blast refers to participants with no history of blast (*n* = 120) and Close blast refers to participants who have exposure to blasts within 10 meters (*n* = 234). Military mTBI (*n* = 247) refers to individuals who experienced at least one mTBI during military service and Non-Military mTBI (*n* = 303) refers to individuals with no mTBI or those who experienced at least one mTBI pre- or post-deployment. No mTBI (*n* = 170) refers to individuals with no history of mTBI, Blunt mTBI (*n* = 224) refers to individuals who experienced at least one blunt-injury related mTBI, and Blast mTBI (*n* = 156) refers to individuals who have experienced at least one blast-injury related mTBI. Significant values reflect Benjamini-Hochberg correction for multiple comparisons. Error bars reflect standard error.

Panel B. Box plot with scatter plot overlay represents log transformed, unadjusted data grouped by blast exposure, military mTBI, and mechanism of injury groups. Error bars reflect the 95% confidence interval.

**Supplemental Figure 4.**

Panel A. Results from Supplemental ANCOVA analyses between blast groups and TBI mechanism groups on residualized values of log transformed NSE, total tau, and IL-6 after controlling for age, sex, and number of blasts or mTBIs. No Blast refers to participants with no history of blast (*n* = 120) and Close Blast (*n* = 234) refers to individuals who have blast exposure between 0 - 10 meters. No mTBI (*n* = 170) refers to individuals with no history of mTBI, Blunt mTBI (*n* = 224) refers to individuals who experienced at least one blunt-injury related mTBI, and Blast mTBI (*n* = 156) refers to individuals who have experienced at least one blast-injury related mTBI. Error bars reflect standard error.

Panel B. Box plot with scatter plot overlay represents log transformed, unadjusted data grouped by blast exposure and blast distance. Error bars reflect the 95% confidence interval.
