# Supplementary material for: Plasma biomarkers associated with deployment trauma and its consequences in post-9/11 era veterans: initial findings from the TRACTS longitudinal cohort
Source: Transl Psychiatry. 2022 Feb 26;12:80. doi: 10.1038/s41398-022-01853-w (PMC8881445; doi:10.1038/s41398-022-01853-w)
Supplement: Supplementary file 2 — Supplemental Tables [file 41398_2022_1853_MOESM2_ESM.docx]

**Summary**

Supplemental Tables & Figures contains four tables and one figure that provide additional details on the exclusion process and missing data. Supplemental Table 4 provides an additional analysis to examine blast exposure at three different blast distances to support our initial close blast analysis.

*Supplemental Table 1.*

*Biomarker Type and Function*

| **Biomarker** | **Type** | **Function** |
| --- | --- | --- |
| Tumor Necrosis Factor alpha (TNFα) | Cytokine | Pro-inflammatory |
| Interleukin 6 (IL-6) | Cytokine | Pro-inflammatory |
| Interleukin 10 (IL-10) | Cytokine | Anti-inflammatory |
| Eotaxin | Chemokine | Coordination of inflammatory cells and allergic inflammation |
| Brain Derived Neurotrophic Factor (BDNF) | Neurotrophin | Neuroprotective |
| Amyloid ß 40 (Aß40) | Peptide | Component of amyloid plaques related to neurodegenerative diseases |
| Amyloid ß 42 (Aß42) | Peptide | Component of amyloid plaques related to neurodegenerative diseases |
| Total Tau | Protein | Marker of microtubule damage |
| Neuron Specific Enolase (NSE) | Enzyme | Marker of axonal damage |
| Neurofilament Light (NfL) | Protein | Marker of axonal damage |
| Phosphorylated Neurofilament Heavy (pNF Heavy) | Protein | Marker of axonal and dendritic damage and degeneration |
| Glial Fibrillary Acidic Protein (GFAP) | Protein | Marker of astrocyte damage |

*Supplemental Table 2*

*Biomarker Missing and Excluded Data*

|  | Missing Complete Sample | Missing Duplicate Sample | CV over 20% | Below LLOQ | Total Missing | Final *n* |
| --- | --- | --- | --- | --- | --- | --- |
| BDNF | 45 | 13 | 5 | 1 | 64 (11.63%) | 486 |
| IL-10 | 40 | 37 | 4 | 0 | 81 (14.73%) | 469 |
| IL-6 | 23 | 54 | 8 | 0 | 85 (15.45%) | 465 |
| TNFα | 29 | 48 | 7 | 0 | 84 (15.27%) | 466 |
| Eotaxin | 31 | 22 | 8 | 0 | 61 (11.09%) | 489 |
| Aß40 | 14 | 27 | 51 | 0 | 92 (16.73%) | 458 |
| Aß42 | 29 | 47 | 20 | 0 | 96 (17.45%) | 454 |
| GFAP | 67 | 40 | 1 | 0 | 108 (19.64%) | 442 |
| NFL | 65 | 40 | 16 | 0 | 121 (22.00% | 429 |
| Tau | 75 | 40 | 64 | 0 | 179 (32.55%) | 371 |
| NSE | 63 | 6 | 17 | 0 | 86 (15.64%) | 464 |
| pNF-H | 38 | 16 | 105 | 3 | 162 (29.45%) | 388 |

*Note.* Missing Complete Sample refers to any data point that was missing due to a participant not providing a blood sample or the amount of blood collected was insufficient to run the assay in duplicate. Missing duplicate sample refers to any data point that did not have two samples available to be run in duplicate, this was either due to a pipetting error or one sample was outside of the assay calibration range. CV over 20% refers to any data point that was excluded because the coefficient of variation was above 20%. Below LLOQ refers to any data point excluded because it was below the lower limit of quantification. Total Missing refers to the total missing data for each biomarker and the percentage missing from the entire sample (*n* = 550). The Final *n* represents the *n* available for analyses.

*Supplemental Table 3*

*Average CV and LLOQ*

|  | Average CV | LLOQ |
| --- | --- | --- |
| BDNF | 3.58% | 0.029 |
| IL-10 | 4.76% | 0.007 |
| IL-6 | 5.12% | 0.011 |
| TNFα | 5.21% | 0.051 |
| Eotaxin | 2.24% | 0.180 |
| Aß40 | 3.94% | 0.675 |
| Aß42 | 5.84% | 0.142 |
| Tau | 7.21% | 0.063 |
| NSE | 2.41% | 9.880 |
| NFL | 6.06% | 0.241 |
| pNF-H | 6.64% | 2.880 |
| GFAP | 3.75% | 0.467 |

*Note.* CV refers to the coefficient of Variation. Average CV was calculated after exclusions for CVs above 20%. LLOQ refers to the lower limit of quantification.

*Supplemental Table 4*

*Relationship between Time, and the number of Blasts and mTBIs, with Plasma Biomarkers*

|  | BDNF | IL-10 | IL-6 | TNFα | Eotaxin | Aß40 | Aß42 | Tau | NSE | NfL | pNF-H | GFAP |
| --- | --- | --- | --- | --- | --- | --- | --- | --- | --- | --- | --- | --- |
| Military TBI | .040 | -.006 | .013 | -.015 | .028 | -.026 | -.011 | -.036 | -.006 | -.039 | -.043 | -.018 |
| Lifetime TBI | .001 | .024 | -.013 | -.028 | .034 | -.003 | -.017 | -.021 | -.011 | .029 | -.018 | -.035 |
| Blast TBI | .068 | .002 | .102 | -.010 | .047 | -.011 | .022 | -.037 | .081 | -.057 | .040 | -.090 |
| Total Blast | -.028 | -.026 | .094 | -.004 | -.009 | -.037 | -.036 | .005 | -.031 | .068 | -.027 | -.003 |
| Close Blast | -.030 | .014 | .005 | -.010 | .029 | -.058 | .002 | .093 | .000 | .057 | -.085 | .016 |
| Mid Blast | -.012 | .008 | .044 | .003 | .006 | -.042 | -.003 | .066 | -.034 | .044 | -.071 | -.017 |
| Far Blast | -.024 | -.034 | .100 | -.003 | -.017 | -.018 | -.042 | -.023 | -.031 | .058 | .009 | -.005 |
| Time since Blast | -.030 | -.045 | -.015 | -.011 | .008 | .010 | -.084 | -.051 | -.002 | -.049 | .121 | -.146 |
| Time since TBI | .041 | -.021 | -.001 | -.023 | .022 | -.201 | -.161 | -.101 | .056 | -.125 | -.013 | -.036 |

*Note.* Partial correlations controlling for age and sex. All correlations reflect Benjamini-Hochberg corrected values.

*Supplemental Table 5*

*Plasma Biomarker Levels Related to Reported Blast Exposure Difference*

|  | No Blast | Mid-Far Blast | Close Blast | *p*-Value | *q*-Value | Pairwise Comparisons |  |
| --- | --- | --- | --- | --- | --- | --- | --- |
|  |  |  |  |  |  |  |  |
|  | *M (SD)* | *M (SD)* | *M (SD)* |  |  |  |  |
| *Neuroprotection* |  |  |  |  |  |  |  |
| BDNF | 7.02 (1.18) | 7.06 (1.20) | 7.25 (1.08) | .115 | .115 | NS |  |
| *Anti-Inflammatory* |  |  |  |  |  |  |  |
| IL-10 | -0.38 (0.50) | -0.31 (0.68) | -0.31 (0.59) | .639 | .639 | NS |  |
| *Pro-Inflammatory* |  |  |  |  |  |  |  |
| IL-6 | 0.22 (0.54) | 0.37 (0.65) | 0.40 (0.72) | .040* | .060 | CB > NB |  |
| TNFα | 0.93 (0.29) | 1.00 (0.37) | 1.02 (0.30) | .185 | .185 | CB > NB |  |
| Eotaxin | 3.64 (0.36) | 3.69 (0.37) | 3.76 (0.35) | .027* | .060 | CB > NB; CB > MB |  |
| *Neurodegeneration* |  |  |  |  |  |  |  |
| Aß40 | 5.36 (0.16) | 5.33 (0.26) | 5.34 (0.17) | .783 | .936 | NS |  |
| Aß42 | 2.07 (0.18) | 2.07 (0.21) | 2.06 (0.22) | .936 | .936 | NS |  |
| Tau | 0.43 (0.58) | 0.36 (0.52) | 0.27 (0.55) | .173 | .346 | NS |  |
| NSE | 9.29 (0.43) | 9.35 (0.51) | 9.42 (0.49) | .173 | .346 | CB > NB |  |
| *Neuronal Damage* |  |  |  |  |  |  |  |
| NfL | 1.73 (0.47) | 1.70 (0.49) | 1.62 (0.50) | .494 | .500 | NS |  |
| pNF-H | 3.10 (0.98) | 3.20 (0.99) | 3.23 (0.97) | .500 | .500 | NS |  |
| *Glial Damage* |  |  |  |  |  |  |  |
| GFAP | 4.22 (0.35) | 4.13 (0.36) | 4.06 (0.37) | .012* | .012* | CB > NB |  |

*Note.* No Blast (NB; *n* = 120) refers to individuals with no history of blast exposure, Mid Blast (MB; *n* = 196) refers to individuals with a history of blast exposure between 11 - 100 meters, and Close Blast (CB; *n* = 234) refers to individuals who have blast exposure between 0 - 10 meters. NS refers to pairwise comparisons that were not significant. *q-*Value represents the Benjamini-Hochberg correction for multiple comparisons. Mean level data represent log transformed concentrations (pg/mL) controlling for age, sex, and number of blast exposures.

*Supplemental Table 6*

*Plasma Biomarker Levels Related to Blast Exposure*

|  | No Blast | Blast | *p -*Value | *q*-Value |
| --- | --- | --- | --- | --- |
|  | *M (SD)* | *M (SD)* |  |  |
| *Neuroprotection* |  |  |  |  |
| BDNF | 7.02 (1.18) | 7.17 (1.14) | .237 | .237 |
| *Anti-Inflammatory* |  |  |  |  |
| IL-10 | -0.38 (0.50) | -0.31 (0.64) | .301 | .301 |
| *Pro-Inflammatory* |  |  |  |  |
| IL-6 | 0.22 (0.54) | 0.38 (0.68) | .030* | .079 |
| TNFa | 0.93 (0.29) | 1.01 (0.33) | .071 | .079 |
| Eotaxin | 3.64 (0.36) | 3.73 (0.36) | .079 | .079 |
| *Neurodegeneration* |  |  |  |  |
| Aß40 | 5.36 (0.16) | 5.34 (0.22) | .658 | .708 |
| Aß42 | 2.07 (0.18) | 2.07 (0.22) | .708 | .708 |
| Tau | 0.43 (0.58) | 0.31 (0.54) | .159 | .318 |
| NSE | 9.29 (0.43) | 9.38 (0.49) | .153 | .318 |
| *Neuronal Damage* |  |  |  |  |
| NfL | 1.73 (0.47) | 1.66 (0.50) | .211 | .288 |
| pNF-H | 3.10 (0.98) | 3.22 (0.97) | .288 | .288 |
| *Glial Damage* |  |  |  |  |
| GFAP | 4.22 (0.35) | 4.08 (0.36) | .004* | .004* |

*Note.* No Blast (*n* = 120) refers to individuals with no history of blast exposure and Blast (*n* = 430) revers to an individual with a history of exposure to at least one blast at any distance range. *q-*Value represents the Benjamini-Hochberg correction for multiple comparisons. Mean level data represent log transformed concentrations (pg/mL) controlling for age, sex, and number of blast exposures.

*Supplemental Table 7*

*Raw Plasma Biomarker Levels Related to Reported Blast Exposure*

|  | No Blast | Mid-Far Blast | Close Blast | *p*-Value | Pairwise Comparisons |  |
| --- | --- | --- | --- | --- | --- | --- |
|  |  |  |  |  |  |  |
| *Neuroprotection* |  |  |  |  |  |  |
| BDNF | 2201.84 (2930.78) | 2425.55 (3677.91) | 2439.93 (2732.81) | .551 | - |  |
| *Anti-Inflammatory* |  |  |  |  |  |  |
| IL-10 | 0.79 (0.62) | 1.43 (7.01) | 1.03 (2.47) | .624 | - |  |
| *Pro-Inflammatory* |  |  |  |  |  |  |
| IL-6 | 1.46 (1.09) | 1.88 (2.01) | 2.11 (3.10) | .110 | CB > NB (*p* = .036) |  |
| TNFα | 2.65 (0.82) | 2.91 (1.15) | 2.91 (0.94) | .106 | CB > NB (*p* = .050) |  |
| Eotaxin | 40.64 (14.90) | 44.72 (36.65) | 45.71 (18.32) | .339 | - |  |
| *Neurodegeneration* |  |  |  |  |  |  |
| Aß40 | 214.88 (36.24) | 212.53 (41.23) | 212.29 (35.76) | .961 | - |  |
| Aß42 | 8.06 (1.43) | 8.11 (1.55) | 8.07 (1.52) | .804 | - |  |
| Tau | 1.82 (1.23) | 1.62 (0.82) | 1.48 (0.70) | .046 | CB < NB (*p* = .013) |  |
| NSE | 12109.76 (7125.73) | 13323.03 (8586.68) | 14181.28 (9297.87) | .179 | *-* |  |
| *Neuronal Damage* |  |  |  |  |  |  |
| NfL | 6.38 (3.82) | 6.30 (3.95) | 5.98 (6.01) | .882 | - |  |
| pNF-H | 37.87 (51.24) | 40.76 (51.27) | 42.30 (57.08) | .635 | - |  |
| *Glial Damage* |  |  |  |  |  |  |
| GFAP | 72.21 (29.79) | 66.02 (24.13) | 61.75 (22.72) | .027 | CB < NB (*p* = .008) |  |

*Note.* No Blast (NB; *n* = 120) refers to individuals with no history of blast exposure, Mid Blast (MB; *n* = 196) refers to individuals with a history of blast exposure between 11 - 100 meters, and Close Blast (CB; *n* = 234) refers to individuals who have blast exposure between 0 - 10 meters. Mean level data represent raw concentrations (pg/mL) controlling for age, sex, and number of blast exposures.

*Supplemental Table 8*

*Raw Plasma Biomarker Levels Related to Military mTBI*

| Non-mTBI | | Military mTBI | |
| --- | --- | --- | --- |
|  | *M (SD)* | *M (SD)* | *p*-value |
| *Neuroprotection* |  |  |  |
| BDNF | 2259.87 (3093.76) | 2531.71 (3184.36) | .219 |
| *Anti-Inflammatory* |  |  |  |
| IL-10 | 1.35 (6.13) | 0.86 (0.78) | .385 |
| *Pro-Inflammatory* |  |  |  |
| IL-6 | 1.82 (2.26) | 1.97 (2.58) | .415 |
| TNFa | 2.85 (0.98) | 2.86 (1.03) | .827 |
| Eotaxin | 43.69 (31.00) | 44.41 (17.79) | .700 |
| *Neurodegeneration* |  |  |  |
| Aß40 | 214.76 (39.36) | 210.58 (35.86) | .457 |
| Aß42 | 8.08 (1.48) | 8.08 (1.54) | .757 |
| Tau | 1.65 (1.01) | 1.55 (0.73) | .948 |
| NSE | 13290.65 (8761.19) | 13624.26 (8532.23) | .355 |
| *Neuronal Damage* |  |  |  |
| NfL | 6.26 (3.80) | 6.09 (5.92) | .911 |
| pNF-H | 43.84 (57.74) | 36.59 (47.43) | .407 |
| *Glial Damage* |  |  |  |
| GFAP | 68.02 (26.71) | 62.66 (22.98) | .022* |

*Note. Note.* Military mTBI (*n* = 247) refers to individuals who experienced at least one mTBI during military service and Non-Military mTBI (*n* = 303) refers to individuals with no mTBI or those who experienced at least one mTBI pre- or post-deployment. Means and standard deviations are reported as raw plasma concentrations (pg/mL) after controlling for age, sex, and number of mTBIs.

*Supplemental Table 9*

*Mean Level Differences in Raw Plasma Biomarkers Related to Mechanism of Injury*

|  | No TBI | Blunt TBI | Blast TBI |  | Pairwise Comparisons |
| --- | --- | --- | --- | --- | --- |
|  | *M (SD)* | *M (SD)* | *M (SD)* | *p*-value |  |
| *Neuroprotection* |  |  |  |  |  |
| BDNF | 2647.77 (3589.42) | 2130.51 (3004.20) | 2461.11 (2794.17) | .416 | - |
| *Anti-Inflammatory* |  |  |  |  |  |
| IL-10 | 1.11 (2.96) | 1.32 (6.55) | 0.87 (0.77) | .715 | - |
| *Pro-Inflammatory* |  |  |  |  |  |
| IL-6 | 1.64 (1.21) | 1.84 (2.48) | 2.21 (3.13) | .059 | Blast mTBI > No mTBI (*p* = .019) |
| TNFa | 2.85 (0.97) | 2.80 (0.97) | 2.94 (1.06) | .422 |  |
| Eotaxin | 45.31 (39.37) | 42.25 (14.96) | 45.25 (19.68) | .415 |  |
| *Neurodegeneration* |  |  |  |  |  |
| Aß40 | 213.23 (36.83) | 212.34 (40.65) | 213.54 (34.96) | .942 | - |
| Aß42 | 8.17 (1.42) | 7.95 (1.55) | 8.17 (1.53) | .317 | - |
| Tau | 1.63 (0.91) | 1.65 (0.99) | 1.52 (0.71) | .561 | - |
| NSE | 13028.97 (7692.81) | 13278.02 (9572.93) | 14093.97 (8259.36) | .314 | - |
| *Neuronal Damage* |  |  |  |  |  |
| NfL | 6.21 (3.58) | 6.14 (3.82) | 6.21 (6.92) | .958 | - |
| pNF-H | 39.91 (52.56) | 42.11 (53.82) | 39.41 (54.69) | .915 | - |
| *Glial Damage* |  |  |  |  |  |
| GFAP | 70.21 (28.37) | 66.16 (24.23) | 60.11 (22.10) | .034* | Blast mTBI < No mTBI (*p* = .011) |

*Note Note.* No mTBI (*n* = 170) refers to individuals with no history of mTBI, Blunt mTBI (*n* = 224) refers to individuals who experienced at least one blunt-injury related mTBI, and Blast mTBI (*n* = 156) refers to individuals who have experienced at least one blast-injury related mTBI. Means and standard deviations are reported raw plasma concentrations (pg/mL) after controlling for age, sex, and number of mTBIs.

*Supplemental Table 10*

*Raw Plasma Biomarker Data by Blast Status in Men and Women*

|  | No Blast | | Mid-Far Blast | | Close Blast | |  |
| --- | --- | --- | --- | --- | --- | --- | --- |
|  |  |  |  |  |  |  |  |
|  | Women | Men | Women | Men | Women | Men |  |
|  | *M (SD)* | *M (SD)* | *M (SD)* | *M (SD)* | *M (SD)* | *M (SD)* |  |
| *Neuroprotection* |  |  |  |  |  |  |  |
| BDNF | 2053.50 (3040.11) | 2232.59 (2925.92) | 3195.39 (5459.16) | 2336.72 (3428.18) | 2800.69 (2909.32) | 2422.16 (2730.22) |  |
| *Anti-Inflammatory* |  |  |  |  |  |  |  |
| IL-10 | 0.74 (0.30) | 0.81 (0.66) | 0.79 (0.50) | 1.51 (7.43) | 0.76 (0.49) | 1.05 (2.54) |  |
| *Pro-Inflammatory* |  |  |  |  |  |  |  |
| IL-6 | 1.24 (0.47) | 1.51 (1.18) | 1.97 (1.25) | 1.87 (2.08) | 3.11 (2.41) | 2.05 (3.13) |  |
| TNFα | 2.23 (0.57) | 2.74 (0.84) | 2.79 (1.76) | 2.93 (1.05) | 2.79 (0.72) | 2.91 (0.95) |  |
| Eotaxin | 33.23 (13.08) | 42.13 (14.86) | 39.51 (12.65) | 44.80 (38.39) | 39.43 (11.85) | 45.95 (18.49) |  |
| *Neurodegeneration* |  |  |  |  |  |  |  |
| Aß40 | 231.43 (30.97) | 211.96 (36.48) | 217.79 (32.44) | 211.94 (42.16) | 209.71 (22.77) | 212.41 (36.26) |  |
| Aß42 | 8.93 (1.35) | 7.91 (1.39) | 8.56 (1.19) | 8.06 (1.58) | 8.38 (1.14) | 8.05 (1.53) |  |
| Tau | 2.04 (1.47) | 1.77 (1.17) | 2.04 (1.02) | 1.56 (0.77) | 1.76 (0.99) | 1.47 (0.68) |  |
| NSE | 10250.14 (7021.37) | 12279.28 (7220.55) | 8447.48 (3164.51) | 13875.59 (8834.51) | 12185.68 (8669.57) | 13987.26 (9454.15) |  |
| *Neuronal Damage* |  |  |  |  |  |  |  |
| NfL | 7.91 (5.79) | 6.05 (3.19) | 6.92 (3.56) | 6.22 (4.00) | 5.37 (1.92) | 6.02 (6.13) |  |
| pNF-H | 33.56 (26.96) | 38.76 (55.04) | 38.13 (39.98) | 41.01 (52.32) | 34.10 (30.02) | 42.69 (58.08) |  |
| *Glial Damage* |  |  |  |  |  |  |  |
| GFAP | 88.75 (32.36) | 68.69 (28.20) | 78.09 (28.07) | 64.55 (23.30) | 62.77 (24.57) | 61.70 (22.70) |  |

*Note.* Women with no blast (*n* = 22); Men with no blast (*n* = 98); Women with mid-far blast (*n* = 22), Men with mid-far blast (*n* = 174); Women with close blast (*n* = 10); Men with close blast (*n* = 224). All mean level data is presented as raw plasma concentrations (pg/mL).

*Supplemental Table 11*

*Raw Plasma Biomarker Data by Military mTBI in Men and Women*

|  | Military TBI | | | |
| --- | --- | --- | --- | --- |
|  | Non-mTBI | | mTBI | |
|  | Women | Men | Women | Men |
| *Neuroprotection* | *M (SD)* | *M (SD)* | *M (SD)* | *M (SD)* |
| BDNF | 1753.39 (2526.94) | 2318.56 (3152.19) | 4060.67 (5524.16) | 2399.39 (2880.08) |
| *Anti-Inflammatory* |  |  |  |  |
| IL-10 | 0.81 (0.45) | 1.42 (6.49) | 0.70 (0.39) | 0.87 (0.81) |
| *Pro-Inflammatory* |  |  |  |  |
| IL-6 | 1.42 (0.89) | 1.87 (2.37) | 2.77 (1.94) | 1.90 (2.63) |
| TNFa | 2.39 (0.77) | 2.91 (0.98) | 2.88 (1.72) | 2.85 (0.95) |
| Eotaxin | 34.62 (11.52) | 44.79 (32.43) | 41.43 (14.61) | 44.62 (18.01) |
| *Neurodegeneration* |  |  |  |  |
| Aß40 | 213.61 (40.05) | 226.13 (30.10) | 210.28 (36.31) | 214.02 (31.07) |
| Aß42 | 8.65 (1.23) | 8.02 (1.49) | 8.69 (1.27) | 8.03 (1.56) |
| Tau | 2.17 (1.31) | 1.57 (0.93) | 1.58 (0.76) | 1.55 (0.73) |
| NSE | 9103.85 (5873.51) | 13594.87 (8952.26) | 11023.05 (6576.65) | 13644.23 (8751.95) |
| *Neuronal Damage* |  |  |  |  |
| NfL | 7.42 (4.83) | 6.09 (3.62) | 6.25 (3.48) | 6.08 (6.08) |
| pNF-H | 37.33 (35.29) | 44.61 (59.85) | 31.21 (24.02) | 37.00 (48.78) |
| *Glial Damage* |  |  |  |  |
| GFAP | 87.74 (30.30) | 65.40 (25.13) | 63.01 (22.92) | 62.63 (23.04) |

Note. Women with no history of military mTBI (*n* = 35); Men with no history military mTBI (*n* = 268); Women with military mTBI (*n* = 19); Men with military mTBI (*n* = 228). All mean level data is presented as raw plasma concentrations (pg/mL).

*Supplemental Table 12*

*Raw Plasma Biomarker Data by Military mTBI in Men and Women*

|  | No TBI | | Blunt TBI | | Blast TBI | |
| --- | --- | --- | --- | --- | --- | --- |
|  | Women | Men | Women | Men | Women | Men |
| *Neuroprotection* | *M (SD)* | *M (SD)* | *M (SD)* | *M (SD)* | *M (SD)* | *M (SD)* |
| BDNF | 1676.80 (2459.21) | 2783.25 (3706.94) | 3536.34 (5539.19) | 1985.91 (2595.31) | 2955.23 (3276.48) | 2428.65 (2770.44) |
| *Anti-Inflammatory* |  |  |  |  |  |  |
| IL-10 | 0.83 (0.36) | 1.15 (3.19) | 0.66 (0.45) | 1.38 (6.87) | 0.84 (0.52) | 0.87 (0.78) |
| *Pro-Inflammatory* |  |  |  |  |  |  |
| IL-6 | 1.55 (0.99) | 1.66 (1.24) | 2.05 (1.86) | 1.82 (2.54) | 2.59 (1.66) | 2.18 (3.21) |
| TNFa | 2.49 (0.88) | 2.90 (0.98) | 2.43 (0.97) | 2.84 (0.96) | 3.12 (2.17) | 2.93 (0.97) |
| Eotaxin | 31.82 (6.40) | 47.47 (41.96) | 41.07 (16.76) | 42.36 (14.82) | 42.22 (10.92) | 45.36 (19.97) |
| *Neurodegeneration* |  |  |  |  |  |  |
| Aß40 | 220.19 (22.13) | 212.42 (38.16) | 223.38 (40.76) | 211.25 (40.59) | 218.90 (21.18) | 214.18 (35.74) |
| Aß42 | 8.92 (1.09) | 8.08 (1.43) | 8.34 (1.38) | 7.91 (1.55) | 8.86 (1.10) | 8.13 (1.55) |
| Tau | 2.19 (1.01) | 1.52 (0.85) | 1.88 (1.50) | 1.62 (0.94) | 1.64 (1.00) | 1.51 (0.69) |
| NSE | 8897.13 (6679.40) | 13279.30 (7802.59) | 11376.74 (7054.48) | 13468.15 (9785.08) | 8757.35 (2446.22) | 14142.64 (8571.59) |
| *Neuronal Damage* |  |  |  |  |  |  |
| NfL | 7.64 (5.14) | 5.95 (3.17) | 6.79 (4.13) | 6.08 (3.80) | 5.92 (2.99) | 6.23 (7.08) |
| pNF-H | 36.52 (29.82) | 40.39 (55.13) | 39.99 (37.09) | 42.33 (55.35) | 17.12 (9.19) | 40.51 (55.77) |
| *Glial Damage* |  |  |  |  |  |  |
| GFAP | 89.65 (24.68) | 66.85 (27.70) | 77.46 (34.27) | 65.07 (22.88) | 56.57 (22.10) | 60.31 (22.17) |

Note. Women with no history of mTBI (*n* = 25); Men with no history of mTBI (*n* = 145); Women with blunt mTBI (*n* = 20); Men with blunt mTBI (*n* = 204); Women with blast mTBI (*n* = 9); Men with blast mTBI (*n* = 147). All mean level data is presented as raw plasma concentrations (pg/mL).
