# Supplementary figures and images for: Plasma biomarkers associated with deployment trauma and its consequences in post-9/11 era veterans: initial findings from the TRACTS longitudinal cohort
Source: Transl Psychiatry. 2022 Feb 26;12:80. doi: 10.1038/s41398-022-01853-w (PMC8881445; doi:10.1038/s41398-022-01853-w)

## Supplemental Figure 1. Participants Exclusions

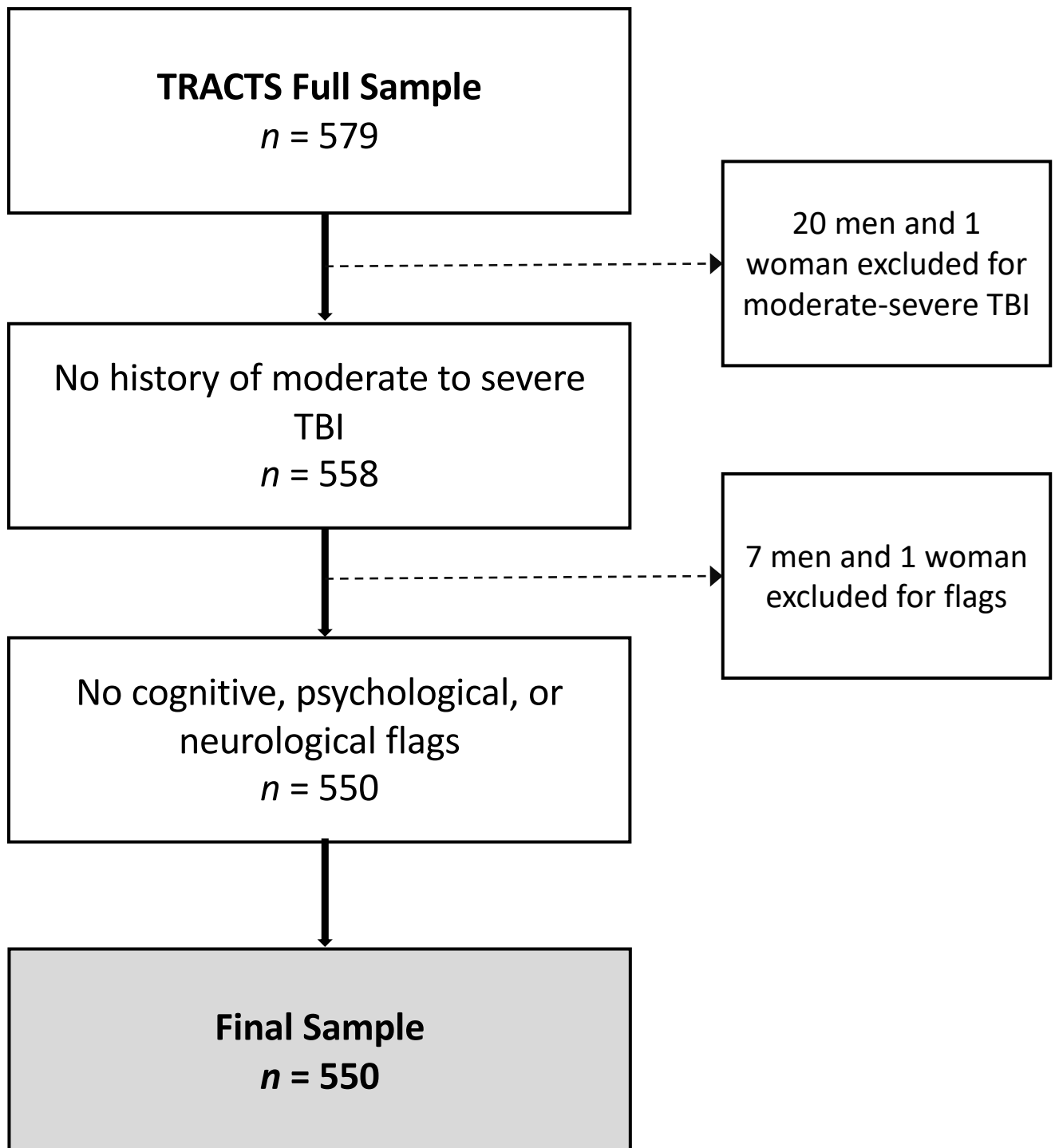

Supplement: Supplementary file 3 — Supplemental Figure 1 [file 41398_2022_1853_MOESM3_ESM.pdf]

Close Blast

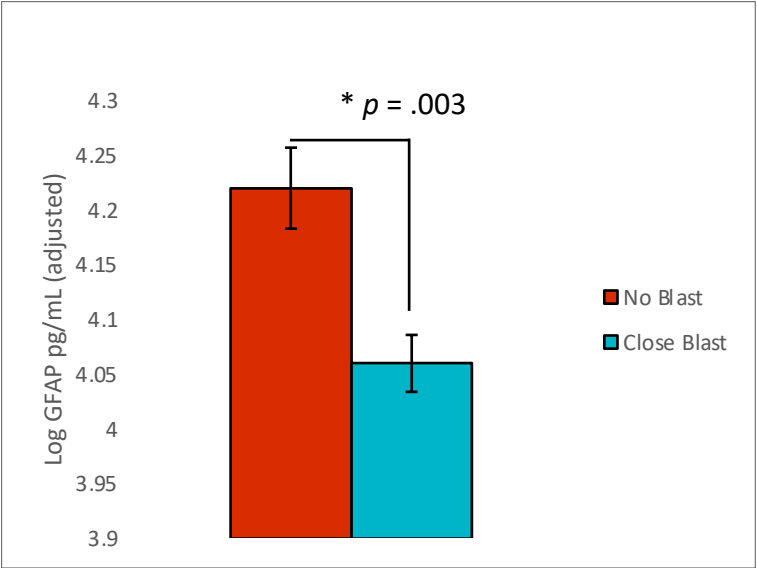

Military mTBI

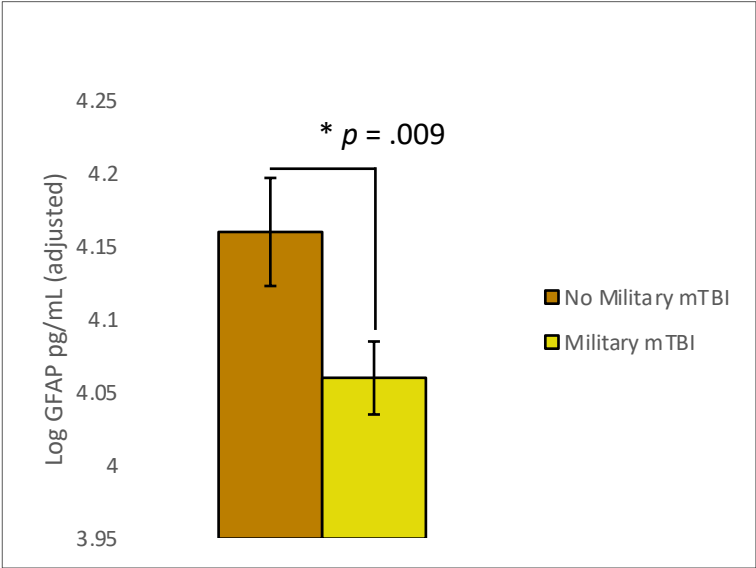

Mechanism of mTBI

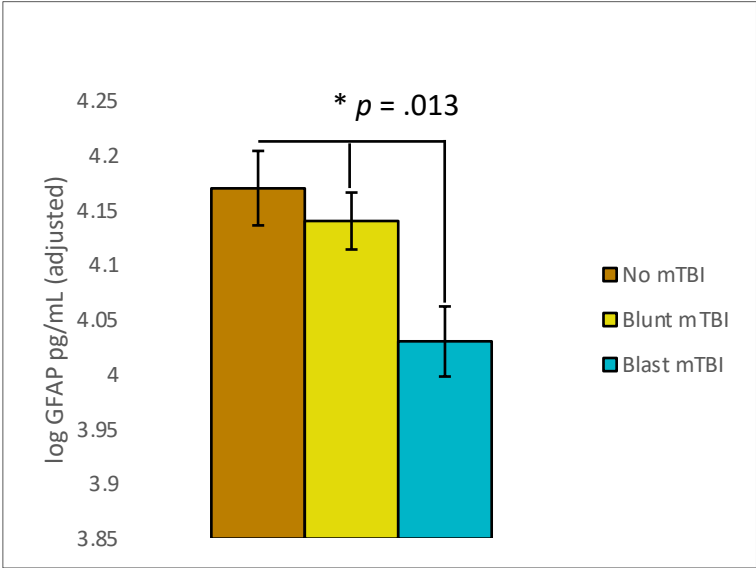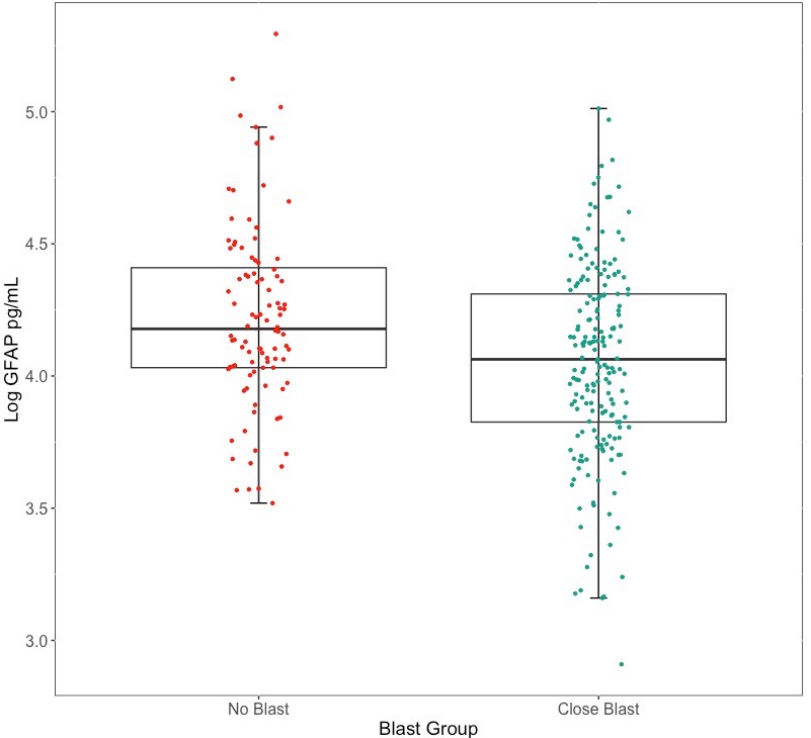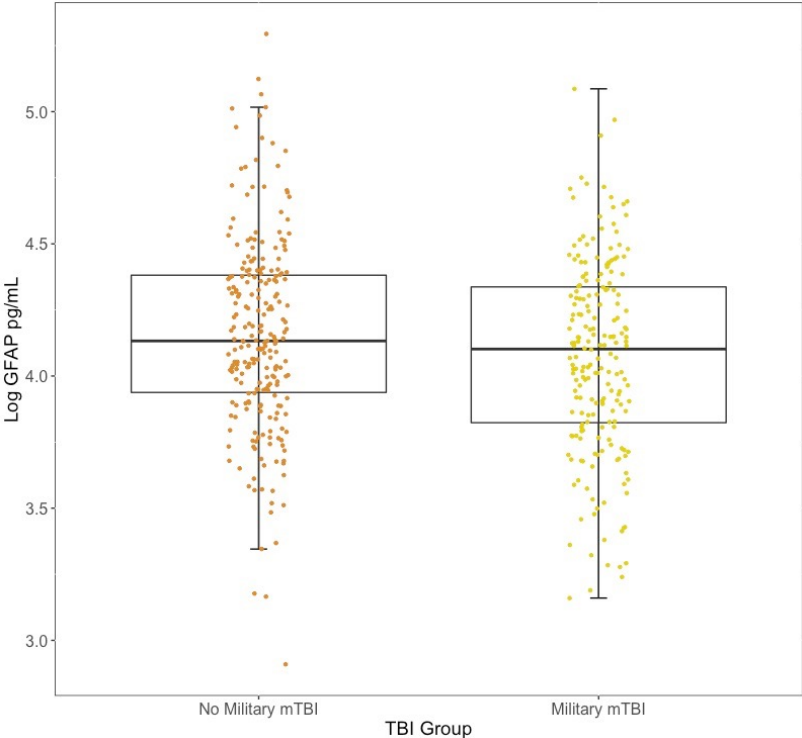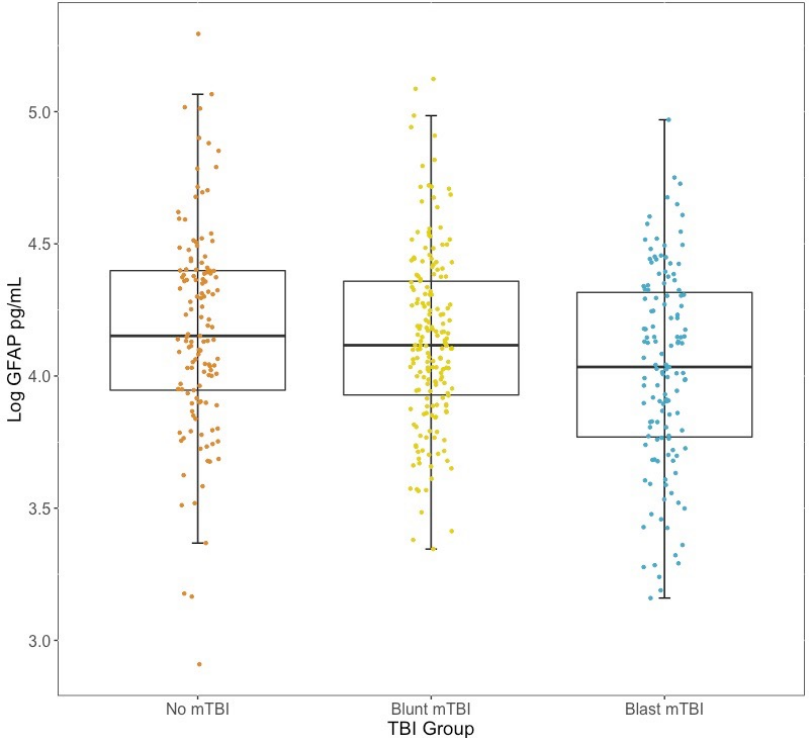

Supplement: Supplementary file 4 — Supplemental Figure 2 [file 41398_2022_1853_MOESM4_ESM.pdf]

IL6

TNF $\alpha$ 

Eotaxin

A

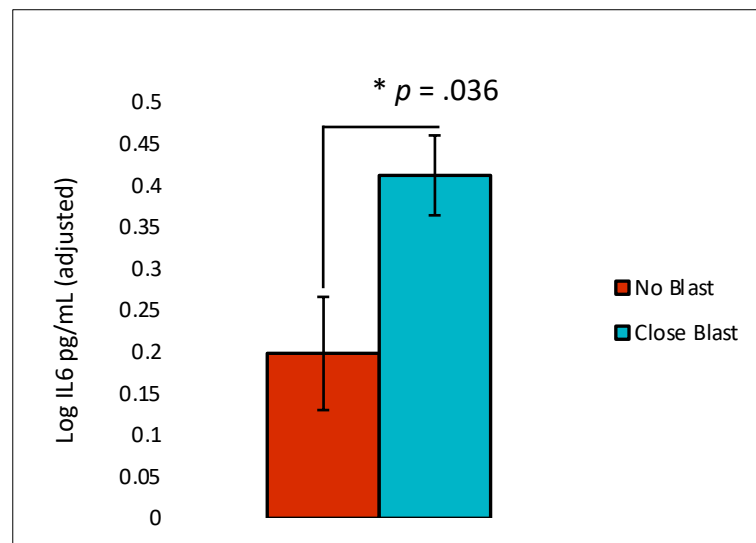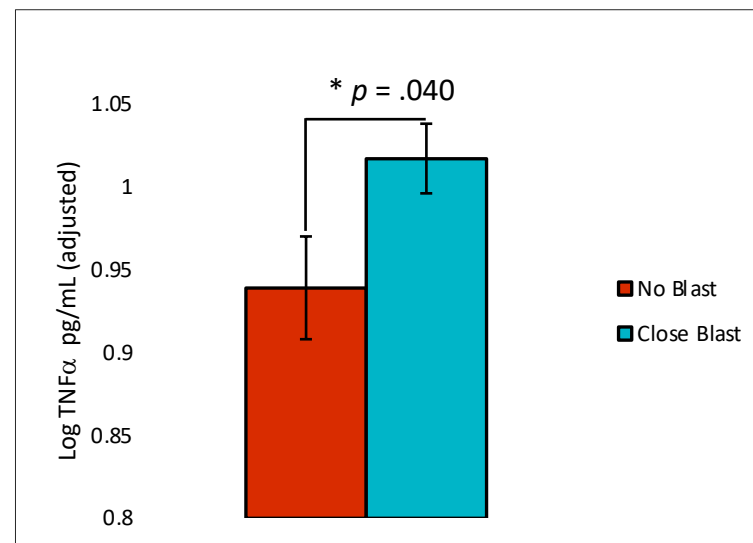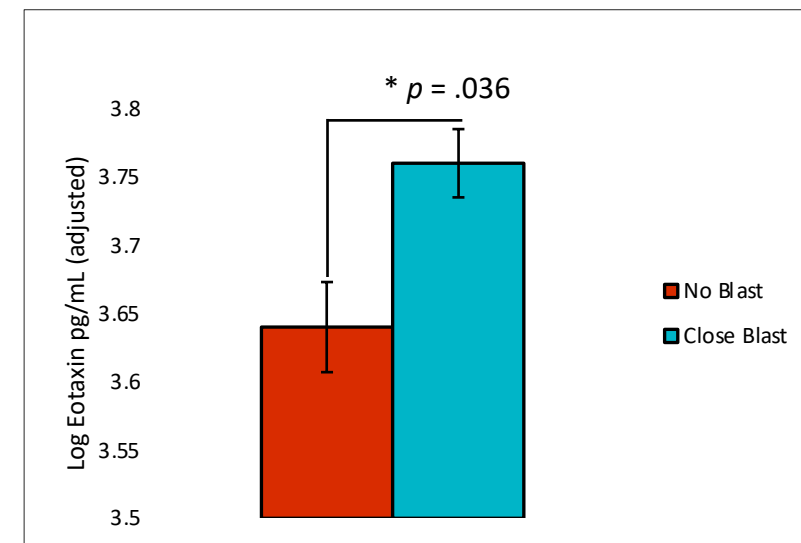

B

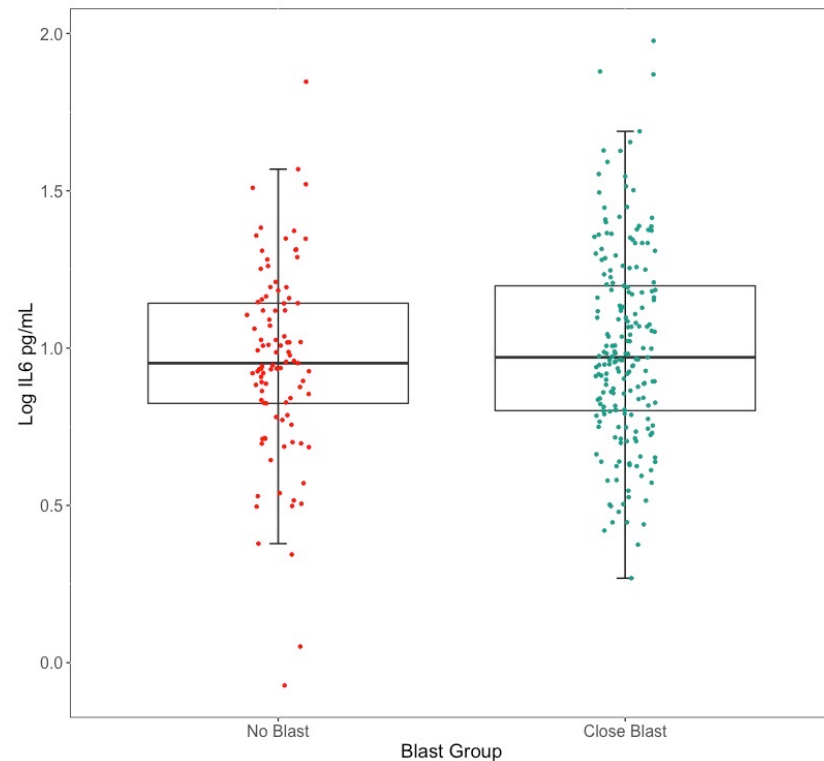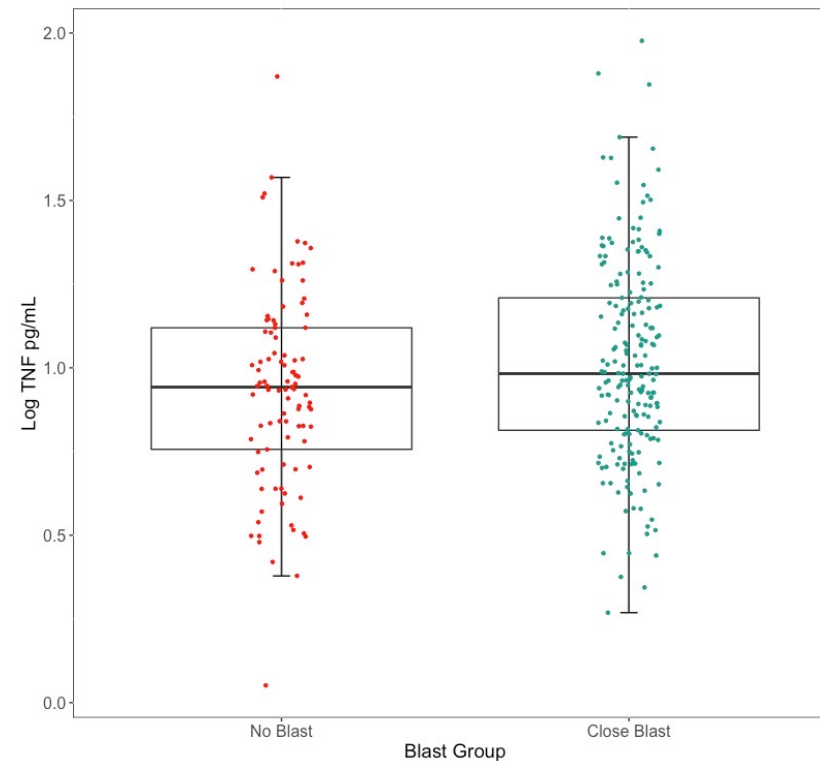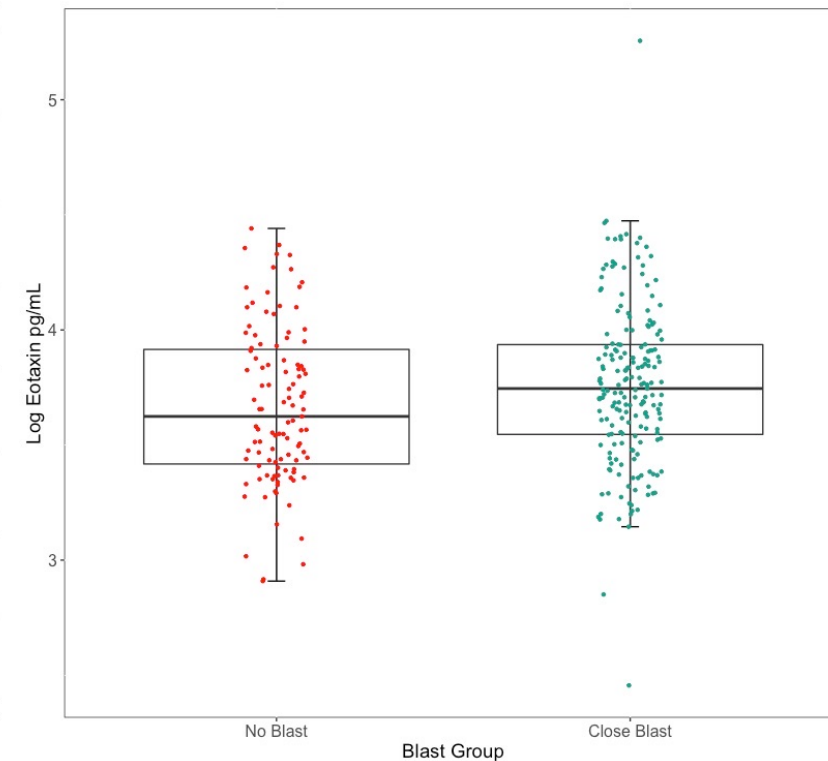

Supplement: Supplementary file 5 — Supplemental Figure 3 [file 41398_2022_1853_MOESM5_ESM.pdf]

# Tau

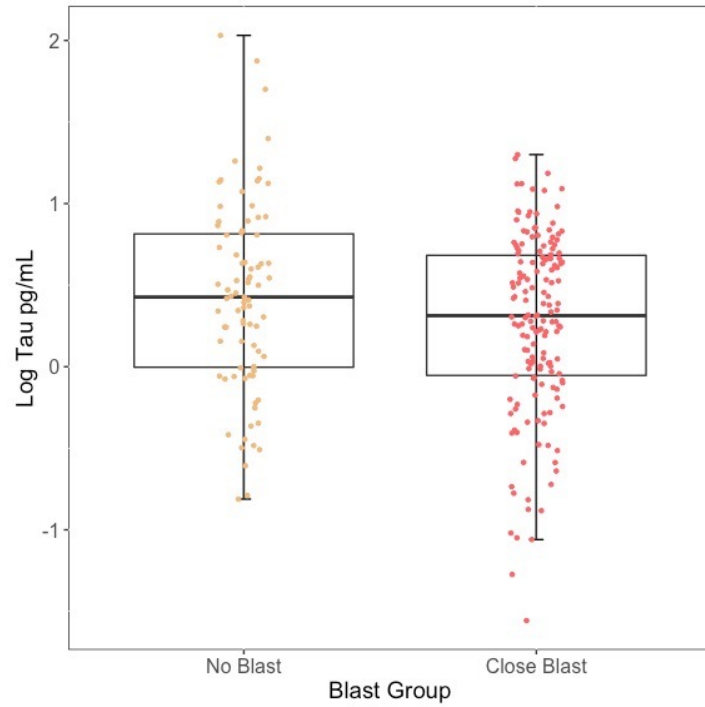

# NSE

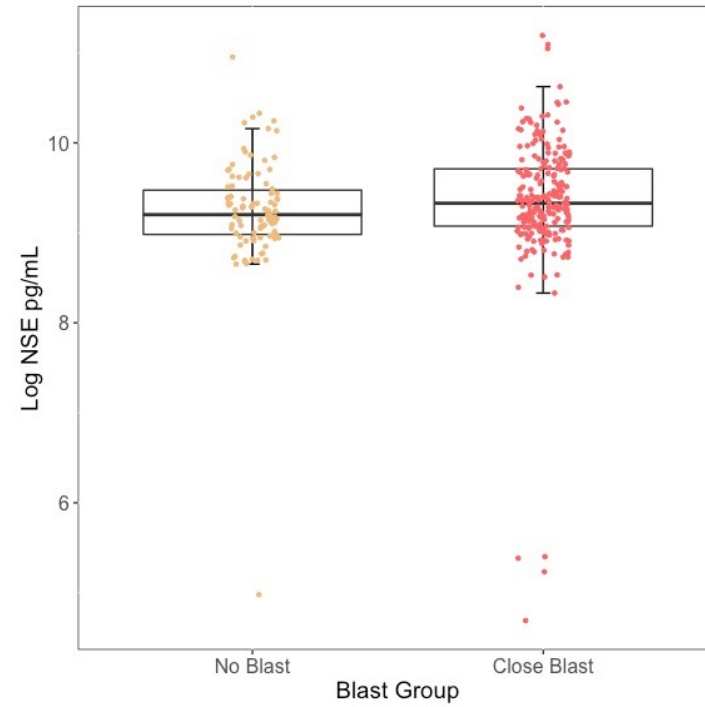

# IL-6

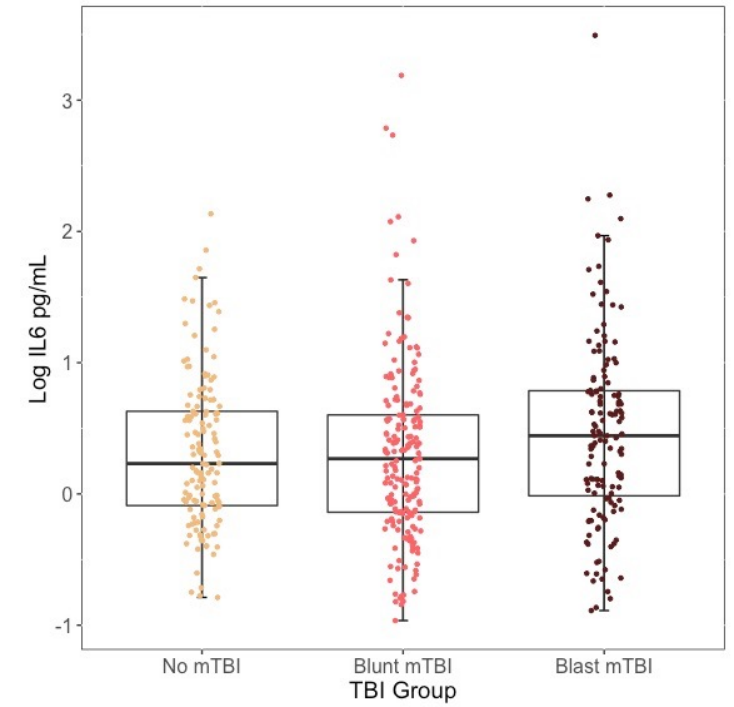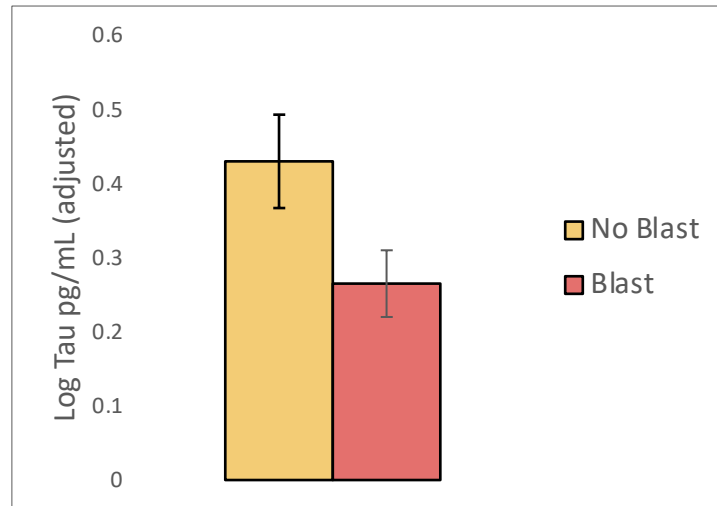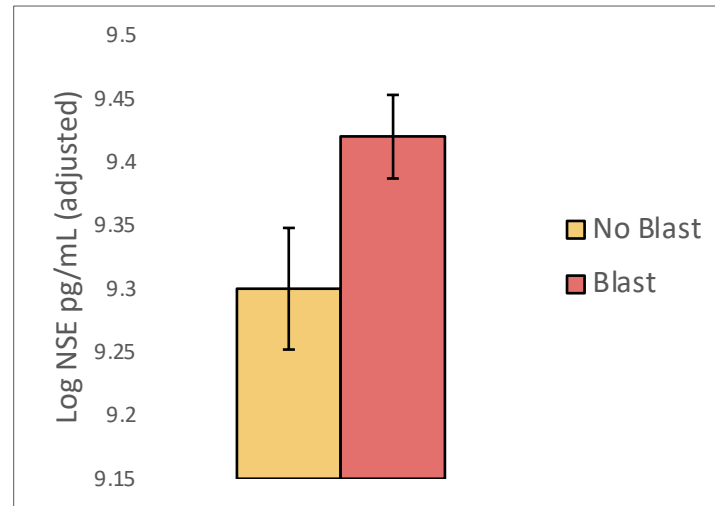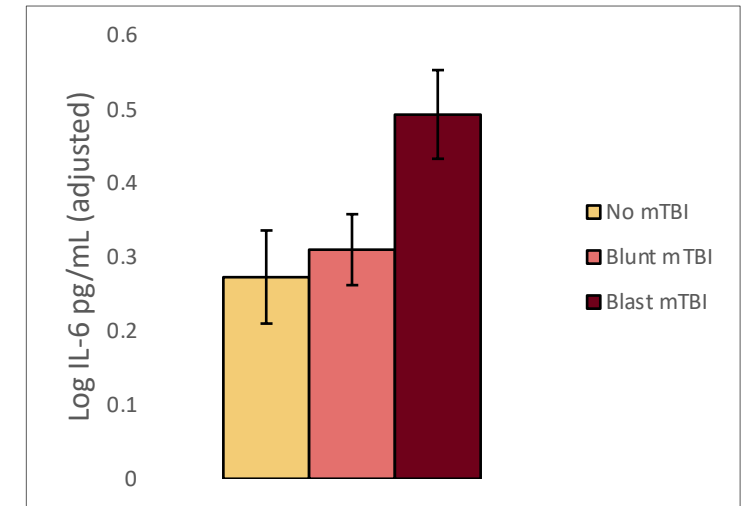

Supplement: Supplementary file 6 — Supplemental Figure 4 [file 41398_2022_1853_MOESM6_ESM.pdf]
